# Contributions of IL-33 in Non-hematopoietic Lung Cells to Obstructive Lung Disease

**DOI:** 10.3389/fimmu.2020.01798

**Published:** 2020-08-13

**Authors:** Li Y. Drake, Y. S. Prakash

**Affiliations:** ^1^Department of Anesthesiology and Perioperative Medicine, Mayo Clinic, Rochester, MN, United States; ^2^Department of Physiology and Biomedical Engineering, Mayo Clinic, Rochester, MN, United States

**Keywords:** IL-33, ST2, inflammation, tissue remodeling, epithelial cells, endothelial cells, airway smooth muscle, fibroblasts

## Abstract

Interleukin (IL)-33 plays important roles in pulmonary immune responses and lung diseases including asthma and chronic obstructive pulmonary disease (COPD). There is substantial interest in identifying and characterizing cellular sources vs. targets of IL-33, and downstream signaling pathways involved in disease pathophysiology. While epithelial and immune cells have largely been the focus, in this review, we summarize current knowledge of expression, induction, and function of IL-33 and its receptor ST2 in non-hematopoietic lung cells in the context of health and disease. Under basal conditions, epithelial cells and endothelial cells are thought to be the primary resident cell types that express high levels of IL-33 and serve as ligand sources compared to mesenchymal cells (smooth muscle cells and fibroblasts). Under inflammatory conditions, IL-33 expression is increased in most non-hematopoietic lung cells, including epithelial, endothelial, and mesenchymal cells. In comparison to its ligand, the receptor ST2 shows low expression levels at baseline but similar to IL-33, ST2 expression is upregulated by inflammation in these non-hematopoietic lung cells which may then participate in chronic inflammation both as sources and autocrine/paracrine targets of IL-33. Downstream effects of IL-33 may occur via direct receptor activation or indirect interactions with the immune system, overall contributing to lung inflammation, airway hyper-responsiveness and remodeling (proliferation and fibrosis). Accordingly from a therapeutic perspective, targeting IL-33 and/or its receptor in non-hematopoietic lung cells becomes relevant.

## Introduction

Interleukin-33 (IL-33) is a member of the IL-1 superfamily. IL-33 is a multifunctional cytokine critically involved in a variety of biological processes such as development and regulation of the immune system, tissue homeostasis vs. repair, and remodeling. IL-33 has been implicated in the pathogenesis of a number of human diseases, including allergy, infection, inflammation, fibrosis, obesity, diabetes, and cancer (1).

**Figure 1:**
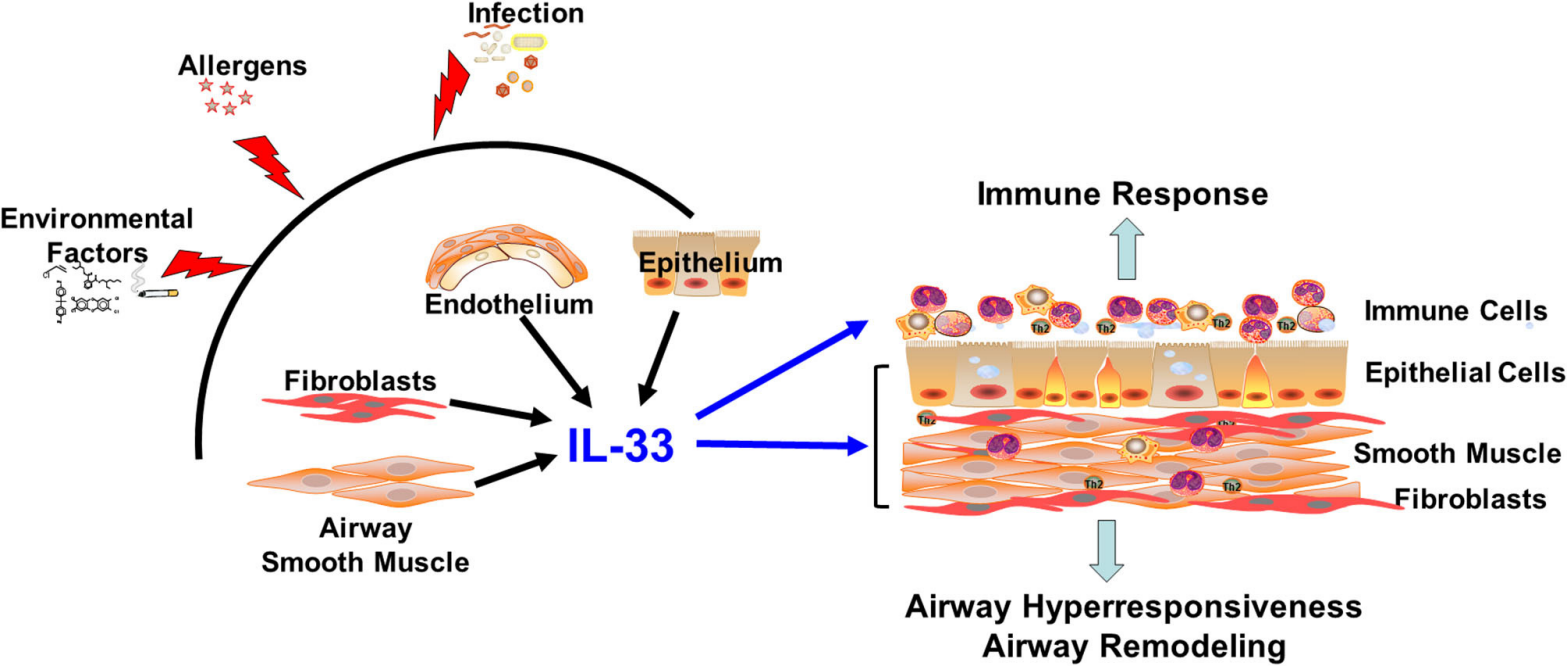
IL-33 in the lung. Infection, allergens, and environmental factors induce IL-33 production and release from resident lung tissue cells. Extracellular IL-33 activates immune cells and non-hematopoietic lung cells to promote immune responses, airway hyperresponsiveness and airway remodeling: aspects relevant to diseases such as asthma.

IL-33 is a tissue-derived nuclear cytokine, produced predominantly by cells of the epithelium and endothelium and by fibroblasts. At baseline, IL-33 is localized to the cell nucleus. Upon cellular stress or injury, IL-33 is released into the extracellular milieu in an active form, and targets cells expressing the IL-33 receptor, commonly known as ST2 (Figure 1). The most recognized function of IL-33 is activation of immune cells involved in type 2 immunity, including group 2 innate lymphoid cells (ILC2s), T helper 2 (Th2) cells, T regulatory cells, macrophages, mast cells, eosinophils, basophils, and dendritic cells (1–3). Extensive studies have analyzed the functional roles of IL-33 in type 2 immunity-associated allergic responses and diseases such as asthma. Multiple genome-wide association studies have identified both the IL-33 and ST2 genes as asthma susceptible loci in humans (4–8). Clinical studies find that IL-33 and ST2 expression are often increased in biological fluids or tissue specimens from patients with allergic diseases (9–12). Preclinical animal data show that the IL-33/ST2 axis is critical in type 2 immune responses (13–17). Beyond type 2 immune cells, recent studies suggest that IL-33 also activates other cell types, such as Th1 cells, natural killer (NK) cells, CD8+ T cells and B cells (18).

Compared with the expansive knowledge regarding IL-33 expression and function in immune cells, there is relatively limited information regarding expression patterns and functional roles of IL-33 in non-hematopoietic lung cells in the context of disease. Accumulating evidence suggests that non-hematopoietic lung cells are not only an important cellular source for IL-33, but also express ST2 and respond to IL-33 stimulation (regardless of source) to participate in lung inflammation and tissue remodeling (Figure 1). In the following sections, we briefly review current knowledge regarding the production and function of IL-33 in non-hematopoietic lung cells, including epithelial cells, endothelial cells, smooth muscle cells, and fibroblasts, in the context of pulmonary inflammation, airway reactivity, and tissue remodeling: aspects critical to the pathophysiology of diseases such as asthma.

## IL-33 Biology

**Figure 2:**
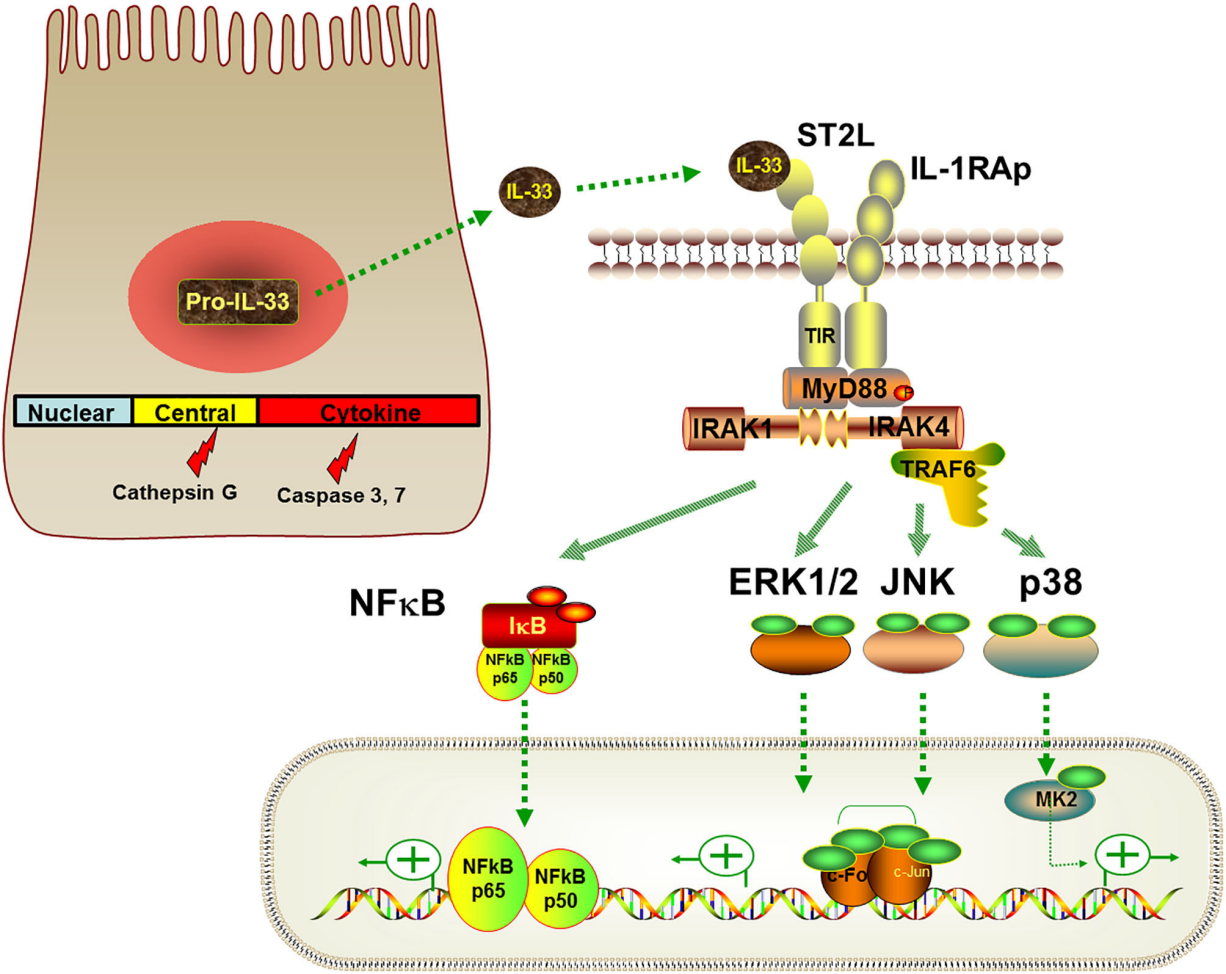
IL-33 signaling pathways. Full-length pro-IL-33 or protease-cleaved IL-33 are released from IL-33 producing cells. Full-length IL-33 consists of a nuclear domain, a central domain and a cytokine domain. Extracellular IL-33 binds to ST2L on target cells, leading to recruitment of the co-receptor IL1RAcP to form an IL-33-ST2L-IL1RAcP complex. After recruiting MyD88, IRAKs and TRAF6, this signaling complex activates NFκB and MAPK family members to induce downstream cellular responses that are cell- and context-dependent.

General IL-33 biology has been described in detail elsewhere (18) and is briefly summarized here in the context of the lung. Human and mouse full-length IL-33 proteins share 55% homology and are 270 and 266 amino acids in length, respectively (19). IL-33 consists of three functional domains: a nuclear domain, a central domain, and an IL-1-like cytokine domain (18) (Figure 2). The nuclear domain contains a chromatin-binding motif that tethers IL-33 protein to chromatin (20). The central domain contains protease recognition sites that allow full-length IL-33 to undergo cleavage into mature bioactive forms encompassing the cytokine domain under inflammatory or stress conditions (21). The IL-1-like cytokine domain binds to ST2 on target cells and mediates the cytokine activities of IL-33 (19, 22).

Under basal conditions, full-length IL-33 protein (about 31 kDa) is localized to the nucleus via the chromatin-binding motif in its nuclear domain (23). The nuclear function of IL-33 is not well understood. In transfected cells, IL-33 appears to regulate chromatin compaction (20) and NF-κB transcriptional activity (24). However, nuclear IL-33 knockdown does not affect either proteome or NF-kB expression in primary human endothelial cells (25). It is currently believed that sequestration of IL-33 in the nucleus of IL-33 producing cells prevents detrimental effects of IL-33 on the host. Supporting this concept, deletion of the chromatin-binding domain of IL-33 in mice results in constitutive IL-33 extracellular release and ST2-dependent lethal eosinophilic and neutrophilic inflammation in multiple organs (26).

IL-33 does not possess a classical signal sequence to direct the cytokine to the endoplasmic reticulum-Golgi secretory pathway. As such, it is not well understood how IL-33 is secreted into the extracellular milieu. Studies suggest that IL-33 can be released from IL-33-producing cells either passively through cell death/damage or actively through cell stress or minor injury (2, 18). IL-33 may be released as either a full-length protein or in processed shorter forms. Several proteases have been shown to cleave IL-33 within its central domain, including serine proteases from neutrophils and mast cells (21, 27) and allergen proteases (28). Although full-length IL-33 displays cytokine activity, the processed forms of IL-33 containing the cytokine domain are functionally 10–30 times more active (27). Caspases have been shown to cleave IL-33 within its cytokine domain and subsequently inactivate IL-33 (29). These studies suggest that proteases play an important role in regulating IL-33 function.

Following extracellular release, IL-33 binds to its receptor ST2 on target cells and regulates cellular function. ST2 is a member of the IL-1 receptor family and has two major isoforms: transmembrane ST2 (ST2L) and soluble ST2 (sST2) (30). ST2L is a membrane bound receptor that binds to and transmits IL-33 signals in target cells. ST2L contains an extracellular domain consisting of three linked immunoglobulin-like motifs, a transmembrane domain, and a cytoplasmic Toll/IL-1 receptor domain. In comparison, sST2 is generated by alternative mRNA splicing and lacks the transmembrane and cytoplasmic domains of ST2L. Rather, sST2 is secreted into the extracellular milieu where it binds IL-33. As such, sST2 may function as a decoy receptor to inhibit IL-33 activity via IL-33 sequestration, thereby preventing IL-33 from binding to ST2L on the cell membrane. Further, whereas basal expression of ST2L is constitutive, sST2 expression is primarily inducible (30). ST2 is predominantly expressed by immune cells, including mast cells, ILC2s, macrophages, dendritic cells, eosinophils, basophils, NK cells, NK T cells, CD4^+^ T cells, and CD8^+^ T cells (2), (18). ST2 is also expressed at low levels by non-hematopoietic lung cells under basal conditions (see sections below). In both immune cells and non-hematopoietic lung cells, ST2 expression is increased by cell activation after pro-inflammatory stimulation (11, 31–33). Enhanced levels of sST2 in biological fluids, such as sputum and serum, have been detected in patients with asthma (9), COPD (34), and idiopathic pulmonary fibrosis (35), and sST2 levels have been shown to correlate with acute exacerbations in these patients (35–37).

IL-33 binds to ST2L with subsequent recruitment of a co-receptor, IL-1 receptor accessory protein (IL1RAcP). In most cell types, this ternary IL-33-ST2L-IL1RAcP complex leads to the recruitment of adaptor proteins including myeloid differentiation primary response protein 88 (MyD88), IL-1R-associated kinases (IRAKs) and tumor necrosis factor (TNF) receptor associated factor-6 (TRAF6) (19, 38). The signaling complex promotes several downstream pathways particularly phosphorylation of inhibitor of κB (IκB)α and mitogen-activated protein kinases (MAPKs) including extracellular signal-regulated kinase (ERK)1/2, p38, and c-Jun N-terminal kinases (JNK) (19). Activation of nuclear factor kappa-light-chain-enhancer of activated B cells (NFκB) and MAPK signaling pathways then drives cellular responses such as proliferation and cytokine production (Figure 2). In addition to activating the MAPK signaling pathway, IL-33 can activate other signaling molecules including phosphoinositide-3-kinase, Janus kinase 2, tyrosine-protein kinase SYK, phosphatases, and GTPases cdc42/Rho (39–41), although these effects are probably cell-type specific and the pathways are not as well-characterized.

At baseline, IL-33 protein is predominantly expressed in non-hematopoietic lung cells but not in immune cells. In humans, bronchial epithelial and endothelial cells are the major IL-33-expressing cell types (10, 42). In mouse lung, IL-33 is mainly expressed by alveolar type II epithelial cells (17). With inflammation or stress, IL-33 expression is broadly upregulated in non-hematopoietic lung cells as well as immune cells (2, 18, 43). Since IL-33 is constitutively and abundantly expressed at basal conditions, IL-33 is thought to function as an “alarmin” cytokine that is released quickly to alert the immune system in response to cellular damage or tissue injury (42). IL-33-deficient mice are fertile and display no gross abnormalities in tissue morphology or development under basal conditions. Upon allergen/antigen exposure, however, IL-33-deficient mice show attenuated airway immune responses (44, 45).

The following sections describe in detail how the IL-33/ST2 axis is regulated specifically in respiratory epithelial cells, endothelial cells, smooth muscle cells and fibroblasts.

## IL-33 and Airway Epithelial Cells

In human airways, nasal, and bronchial epithelial cells are the primary cell types expressing IL-33 at baseline (10, 46). IL-33 expression in these cell types is substantially increased in patients with allergic asthma, allergic rhinitis, aspirin-exacerbated respiratory disease, and COPD (10, 47–50). In healthy bronchial epithelium, IL-33 is localized primarily to basal cells of the epithelial tract, but expression becomes more widespread in asthmatic bronchial epithelium (10). Moreover, allergen airway challenge further increases IL-33 expression in bronchial epithelium of asthmatic patients (51). In COPD, lung IL-33 expression is increased specifically in a subset of epithelial progenitor basal cells present in regions of epithelial hyperplasia and mucous cell remodeling (50). In contrast to humans, mouse bronchial epithelial cells do not express IL-33 at baseline, while alveolar type II epithelial cells (type 2 pneumocytes) are the major sources of IL-33 (17). Alveolar epithelial IL-33 is upregulated by a number of pathologic stimuli including nematode or viral infections, and exposure to cigarette smoke, ozone, cysteine proteases, uric acid, bleomycin, or allergens (15, 52–56). Moreover, with viral infection or cigarette smoke exposure, mouse bronchial epithelial cells start to express IL-33 (50, 54).

In addition to being sources of IL-33, human airway epithelial cells also express ST2 (57). Interestingly, ST2 expression is extremely low at baseline, but is significantly increased in nasal epithelium of allergic rhinitis patients (47), and in bronchial epithelium of severe asthma patients (58). In mouse, baseline ST2 expression in alveolar type II epithelial cells is detectable by flow cytometry (43).

The presence of ST2 in airway epithelial cells suggests autocrine effects such that IL-33 can self-regulate its signaling. Indeed, IL-33 stimulation of human airway epithelial cells *in vitro* induces IL-33 mRNA upregulation, although ST2 expression is not affected (11, 57). In mouse models, airway administration of IL-33 enhances both IL-33 and ST2 expression in lung epithelial cells (11, 43, 59). Moreover, IL-33 and IL-13 work synergistically to enhance IL-33 expression in bronchial epithelial cells (11).

In addition to self-regulation, IL-33 induces production of inflammatory cytokines including IL-8, granulocyte-macrophage colony-stimulating factor and IL-17F in human nasal and bronchial epithelial cells, acting via MAPKs (47, 57, 60, 61). Notably, type 2 immunity-associated cytokines, such as IL-4, IL-5, IL-10, and IL-13, are not induced in epithelial cells by IL-33 under the same culture conditions. IL-33 also has a role in promoting rhinovirus-induced chemokine CXCL10 production in human bronchial epithelial cells (62).

A known prominent function of IL-33 is to induce immune cells to secrete type 2 cytokines such as IL-5 and IL-13 (1–3). IL-13 is a strong inducer of both IL-33 and ST2 mRNA expression in human bronchial epithelial cells. Moreover, IL-33 and IL-13 work synergistically to enhance IL-33 expression in bronchial epithelial cells (11). Based on these observations, it has been suggested that IL-33 and type 2 cytokines can form feed-forward circuits to sustain lung inflammation (11).

IL-33 also plays a role in epithelial repair and remodeling following injury. In influenza infection mouse models, IL-33 promotes production of amphiregulin, a tissue remodeling-associated protein, by innate lymphoid cells and T regulatory cells to maintain epithelial integrity (63, 64). Although airway epithelial cells have been shown to produce amphiregulin (65), it is unknown whether IL-33 can induce amphiregulin in these cells. The IL-33/ST2 axis also seems to have a protective effect in ozone-induced lung injury where tight junction protein reductions are exaggerated in IL-33- or ST2-deficient mice (53). However, it is unknown whether the protective effect of IL-33 occurs through direct stimulation of lung epithelial cells or via indirect influences of surrounding cells.

In summary, given that the airway epithelium is the first line of defense, predominant expression in this layer makes IL-33 an important cytokine for initiation and modulation of immune responses to environmental pathogens or insults (2, 3, 18). Although limited information is available, the effects of IL-33 on airway epithelial cells *per se* are likely important in chronic lung inflammation. With ST2 expression in epithelial cells upregulated during chronic lung inflammation, IL-33 may amplify inflammatory responses. Moreover, IL-33 is critically involved in the maintenance of epithelium integrity after respiratory insults, although the direct IL-33 effects on airway epithelial cells in this process are not well-understood.

## IL-33 and Endothelial Cells In The Lung

In humans, IL-33 is abundantly expressed in healthy vascular endothelium of large and small blood vessels (42, 66). In contrast, IL-33 is generally not expressed in the mouse vascular endothelium at baseline, as demonstrated in an Il-33–LacZ gene trap reporter mouse strain (67). ST2 expression in lung endothelium has not been well-studied. One report indicated that healthy pulmonary vascular endothelium expresses ST2 protein at very low levels in both mouse and human (68). Additional work has shown that IL-33 and ST2 expression in pulmonary vascular endothelium is increased under certain pathological conditions, such as bronchiectasis and pulmonary hypertension in humans (68), allergen airway challenge in asthmatic patients (51), and after *in vivo* exposure to hypoxia or cigarette smoke in mice (68, 69). Interestingly, IL-33 expression is markedly decreased in blood vessels of patients with idiopathic pulmonary arterial hypertension (70), suggesting that IL-33 plays a dynamic role in lung diseases.

Besides vascular endothelial cells, some lymphatic endothelial cells in the lung also express IL-33 under inflammatory conditions. IL-33^+^ lymphatic endothelial cells have been found in mouse lungs after ovalbumin (OVA) exposure and in nasal polyps of patients with eosinophilic chronic rhinosinusitis. These cells seem to play an important role in maintenance of memory Th2 cells in chronic allergic airway inflammation (71).

Much information regarding IL-33 biology in endothelial cells is derived from *in vitro* studies using isolated human primary endothelial cells, including human pulmonary microvascular and arterial endothelial cells, and human umbilical vein endothelial cells (HUVECs). Although not lung-derived, HUVECs have been widely used for IL-33 studies and are briefly reviewed here. HUVECs express both IL-33 and ST2, with expression associated with cell proliferation (57, 66). IL-33 is generally expressed in resting non-proliferative HUVECs whereas ST2 is preferentially expressed in non-quiescent proliferating HUVECs (66, 72, 73). IL-33 expression in HUVECs can be induced by Notch-signaling and inhibited by treatment with TNF, IL-1β or vascular endothelial growth factor (VEGF) (66, 72). Using a global proteomic approach, IL-33 stimulation of HUVECs has been found to induce the expression of many inflammatory proteins, including cell adhesion receptors involved in leukocyte/endothelium interactions, inflammatory chemokines and cytokines, proteins involved in antigen processing and presentation, and NFκB-signaling molecules (25). Several other studies have shown that IL-33 stimulation of HUVECs induces upregulation of nitric oxide, IL-6, IL-8, monocyte chemoattractant protein-1, CXCL1, granulocyte-macrophage colony-stimulating factor and macrophage colony-stimulating factor, tissue factor, E-selectin, intercellular adhesion molecule-1, and vascular cell adhesion molecule-1 (73–81). Functionally, IL-33 stimulation of HUVECs induces cell proliferation, migration and microvessel formation (76, 82). IL-33 also decreases cell integrity and increases barrier permeability in HUVECs (76, 83). These findings suggest that IL-33 may play an important role in both angiogenesis and in endothelial barrier function.

A limited number of studies have explored the IL-33/ST2 pathway in human pulmonary vascular endothelial cells. These studies have shown that such cells express both IL-33 and ST2, and that their expression is increased by IL-4 treatment and by pathological conditions such as hypoxia (57, 68). In response to IL-33 stimulation, human pulmonary vascular endothelial cells produce IL-6, IL-8 and monocyte chemoattractant protein-1 via activation of ERK and p38 MAPK pathways (57). IL-33 stimulation also enhances cell proliferation, adhesion, spontaneous angiogenesis, and expression of remodeling-associated proteins hypoxia-inducible factor-1α, VEGFA, and VEGF receptor-2 (68).

Several *in vivo* mouse studies suggest that IL-33 plays an important role in pulmonary vascular remodeling during chronic airway inflammation. Long-term IL-33 airway administration induces pulmonary arterial hypertrophy in mice that is dependent on IL-5-producing ILC2s and eosinophils (84). It is unknown whether the direct action of IL-33 on endothelial cells contributes to development of pulmonary arterial hypertrophy in this model. Using a similar mouse model, another group found that chronic IL-33 airway administration induces angiogenesis in the lung with increased expression of angiogenic factors, including amphiregulin, angiogenin, endothelin-1, epidermal growth factor, and insulin-like growth factor-1 (82). IL-33 has also been shown to promote vascular remodeling in hypoxic pulmonary hypertension (68). Administration of IL-33 *in vivo* exacerbates hypoxia-induced pathological changes associated with pulmonary hypertension in wild type mice. Conversely, hypoxia-induced pathological changes are diminished in ST2^−/−^ mice. Hypoxia increases IL-33/ST2 expression in human pulmonary arterial endothelial cells both *in vivo* and *in vitro*. Knockdown of either the *Il33* or *st2* genes attenuates hypoxia-induced adhesion and tubule formation in human pulmonary arterial endothelial cells (68). Thus, hypoxia-induced IL-33/ST2 upregulation may form a positive feedback loop to drive chronic vascular remodeling in the lung.

In summary, pulmonary vascular endothelium is one of the major cell sources for IL-33 production in the human lung. Endothelial cells may play important roles in the early responses against pathogens circulating in the blood stream by releasing IL-33 to alarm the immune system. Pulmonary endothelial cells also can respond to IL-33 to promote pulmonary inflammation and tissue remodeling, likely in autocrine and paracrine manners.

## IL-33 and Airway Smooth Muscle (ASM) Cells

Human ASM cells have been found to express IL-33 mRNA at relatively high levels *in vitro* (19). However, ASM bundles in healthy human bronchial biopsies show little to no IL-33 protein expression (85, 86). IL-33 protein expression in ASM bundles is significantly increased in asthmatic patients compared with healthy controls (85, 86). In primary human ASM cells, IL-33 mRNA expression is enhanced by *in vitro* inflammatory stimulation, such as TNF-α, interferon-γ, double stranded RNA, ATP, and rhinovirus infection (85, 87). Both human and mouse ASM cells have been shown to express very little ST2 protein at steady-state, with expression increased by IL-33 stimulation *in vitro* or intranasal exposure to OVA *in vivo* (86, 88).

Mouse experiments suggest that IL-33 is involved in development of airway hyperresponsiveness (AHR), a hallmark feature of asthma. Intranasal IL-33 administration induces AHR in mice (12, 86, 89). Conversely, ST2-deficient mice show a severely compromised allergen-induced airway resistance response (12, 89). Since ASM cells play critical roles in AHR (90), the direct action of IL-33 on ASM cells has been investigated *in vitro*. IL-33 stimulation induces calcium influx in ASM cells, suggesting increased contractility (86). However, this idea is supported by some, but not all, studies. First, IL-33-pretreated human ASM cells contract similarly to untreated ASM cells in collagen gels (86). Second, IL-33 does not cause bronchoconstriction in mouse precision-cut lung slices (PCLS) (86). Third, pretreatment of mouse PCLS with IL-33 has no effect on cholinergic agonist carbachol-induced luminal diameter changes (86). In contrast to these findings, pretreatment of mouse PCLS with IL-33 has been found to increase methacholine-induced luminal contraction, with this effect absent in PCLS from mice deficient in IL-4, IL-5, IL-9, or IL-13 (89). Since IL-13 can directly stimulate airway contraction (91) and IL-33-induced AHR is IL-13-dependent (12, 86, 89), IL-33 likely induces AHR *in vivo* by stimulating IL-13 production in immune cells, such as ILC2s and mast cells, and subsequently IL-13 promotes increased airway contraction (12, 86, 89), i.e., an indirect effect of IL-33. It remains unclear whether the direct action of IL-33 on ASM cells has a role in AHR.

IL-33 has also been shown to be involved in ASM remodeling and wound healing. Long-term IL-33 intranasal administration induces ASM hypertrophy and hyperplasia in mice (92). IL-33 also increases smooth muscle thickening in OVA-sensitized mice (93). However, an *in vitro* study showed that IL-33 does not affect proliferation or survival of primary human ASM cells, suggesting that IL-33 may regulate ASM remodeling by an indirect mechanism *in vivo* (86). IL-33 seems to have a role in ASM wound repair as shown in studies using an IL-33-neutralizing antibody following mechanical injury *in vitro* (86), although the underlying mechanism for this effect remains unknown.

ASM cells can produce a broad class of chemokines/cytokines, eicosanoids, and prostaglandins under inflammatory conditions (94). However, it is largely unknown whether IL-33 stimulates ASM cells to produce these molecules. So far, only one study has shown that IL-33 induces keratinocyte-derived chemokine production in murine ASM cells *in vitro* (88).

In summary, current evidence suggests that IL-33 plays a role in AHR development, ASM remodeling and wound healing. However, many of these effects appear to be mediated by IL-33 indirectly regulating ASM cells via immune cells, such as mast cells and ILC2s. The detection of increased IL-33 expression in asthmatic ASM cells indicates that ASM cells may serve as an important cell source for IL-33 under inflammatory conditions. How this increased IL-33 expression in asthmatic ASM cells affects airway structure or function requires further study.

## IL-33 and Lung Fibroblasts

Historically, ST2 was first identified in 1989 as a serum-responsive protein in murine fibroblast BALB/c-3T3 cells (95). Later studies found that lung fibroblasts express both IL-33 and ST2. At baseline, IL-33 and ST2 mRNA and protein expression levels are very low in both human and mouse lung fibroblasts (19, 96, 97). IL-33 expression in lung fibroblasts can be upregulated *in vitro* by stimulation with TNF-α, TNF superfamily member 14 (also known as LIGHT), IL-1β, and second messenger cyclic GMP-AMP (19, 97, 98) and downregulated by interferon-γ (99). *In vivo*, IL-33 expression in lung fibroblasts is increased in mice with bleomycin-induced pulmonary fibrosis (56), and in patients with idiopathic pulmonary fibrosis (100). Similar to IL-33 expression, ST2 expression in lung fibroblasts is upregulated *in vitro* by stimulation with inflammatory cytokines, including IL-1β, IL-4, IL-13, and TNF-α (96, 101) and by OVA airway administration *in vivo* (96).

Extensive studies have demonstrated that IL-33 plays an important role in lung tissue remodeling and fibrosis (102, 103). In mouse models, IL-33 intranasal administration induces deposition of extracellular matrix proteins in the lung, including collagen I, III, V and fibronectin, and increased expression of fibrosis-associated molecules, including connective tissue growth factor and fibroblast growth factor receptor 4 (12, 104). IL-33 airway administration also potentiates bleomycin-induced lung fibrotic changes in mice (56, 100). In contrast, ST2 deficiency or anti-IL-33 antibody treatment attenuates lung tissue remodeling after intranasal exposure to bleomycin, house dust mite, OVA, or influenza virus (12, 56, 63, 104, 105). Proposed mechanisms by which IL-33 regulates lung tissue remodeling include activation of several cell types, including macrophages, T regulatory cells and ILC2s, to produce tissue remodeling-associated factors such as amphiregulin, IL-13 and transforming growth factor-β1 (56, 63, 64) with these factors activating lung fibroblasts and other tissue cells to promote tissue repair and remodeling.

Considering that lung fibroblasts upregulate ST2 expression under inflammatory conditions, IL-33 may directly stimulate lung fibroblasts to promote lung tissue remodeling in an autocrine/paracrine fashion. For example, IL-33 stimulation of primary human or murine lung fibroblasts increases cell proliferation and production of tissue remodeling-associated factors, including collagen I, III, IV, fibronectin 1, matrix metallopeptidase 9, tissue inhibitor of matrix metalloproteinases 1, TRAF6, NFκB, α-smooth muscle actin, and eotaxin (104, 106–108). Interestingly, IL-33 stimulation increases collagen production only in lung fibroblasts from children with severe therapy-resistant asthma but not in lung fibroblasts from healthy adults (12). These results suggest that IL-33 may directly activate lung fibroblasts to participate in the tissue remodeling/fibrosis process, at least under inflammatory conditions.

In summary, lung fibroblasts express very low levels of IL-33 and ST2 at steady state and the expression levels are increased under inflammatory conditions. IL-33 possibly regulates lung tissue remodeling and fibrosis by both direct and indirect activation of lung fibroblasts.

## Conclusions and Perspectives

Accumulating evidence suggests that non-hematopoietic lung cells play important roles in IL-33-mediated biological responses. At baseline, epithelial cells, and endothelial cells appear to be the predominant cellular sources of IL-33 in the lung, with generally low levels of ST2 expression. Thus, the main function of non-hematopoietic lung cells appears to be to serve as producers of IL-33, rather than as responders, in the early response phase (acute response) to any pathologic stimulation. Under either inflammatory or stress conditions, however, the expression of both IL-33 and ST2 is increased in non-hematopoietic lung cells, a response which may amplify IL-33/ST2 signaling in an autocrine and/or paracrine manner, ultimately leading to amplification of chronic airway responses. Thus, non-hematopoietic lung cells likely participate in chronic airway responses as both producers of, and responders to, IL-33.

IL-33 functions in both immune cells and non-hematopoietic lung cells. Although the roles of IL-33 in regulating immunity have been extensively studied, many questions remain to be answered regarding how IL-33 regulates non-hematopoietic lung cells. Available *in vitro* data suggest that IL-33 can directly promote cell proliferation and production of tissue remodeling factors in non-hematopoietic lung cells. However, it is unknown whether this occurs *in vivo*. The importance of IL-33 effects on non-hematopoietic lung cells in disease processes therefore needs to be determined. It is expected that studies with tissue- or cell-specific ST2 knock-out mice will facilitate answering these questions. Such tools have been successfully used to discern the functional roles of IL-33 and ST2 in the cardiac response to pressure overload (109), but there is currently no data in the lung tissue cells. However, these mouse studies may need to be cautiously interpreted due to species differences in IL-33 and ST2 expression, particularly mouse vs. human. Considering that IL-33 is a strong activator of immune cells, it is possible that many IL-33 effects are mediated through cross-talk between immune cells and non-hematopoietic lung cells *in vivo*. Cross-talk between lung epithelial cells and immune cells through IL-33 and IL-13 has been suggested to be important in chronic lung inflammation (11). Future studies are required to identify cross-talk signaling cascades between immune cells and other types of non-hematopoietic lung cells in chronic lung inflammation and tissue remodeling.

As a multifunctional cytokine, IL-33 appears to have both beneficial and pathological roles in lung responses to pathogens or insults. While IL-33 can cause inflammation-induced airway tissue damage, it can also promote tissue repair after injury. The underlying mechanisms for both of these responses appear to involve immune cells as well as non-hematopoietic lung cells. Existing evidence suggests that IL-33 may engage in different signaling pathways in different cell types (110), and thus understanding how IL-33 dictates beneficial vs. pathological responses may necessitate identifying differential signaling pathways. From a therapeutic perspective, identification of detrimental signaling cascades and targets becomes important. Some important additional questions which remain to be answered are: (1) How do different co-stimulation signals (environmental cues) modify the final outcomes of IL-33-mediated cellular responses; (2) How does IL-33 or receptor expression and downstream signaling differ between cell types, and how are they modulated by natural factors such as age or sex; (3) Relevant to sex, is there a role for sex steroids in modulating IL-33 biology, or vice versa, toward explaining sex differences in diseases such as asthma or COPD (4, 111) What are the factors that modulate IL-33 biology in airway disease, and how do such effects change with the duration and/or extent of disease given IL-33's role as an alarmin?

Evidence that the IL-33/ST2 pathway plays a crucial role in the development of asthma and other lung diseases has resulted in investigations to determine whether targeting this pathway is of therapeutic value. Reagents have been developed to block the binding of IL-33 to ST2, including anti-IL-33 and anti-ST2 receptor antibodies and soluble decoy receptors, with several clinical trials for patients with asthma and COPD currently underway (112, 113). Given that IL-33 has both beneficial and pathological roles in lung responses, our ability to better understand the mechanisms of these complex IL-33 functions will ultimately benefit future efforts to develop more effective and targeted strategies for the treatment of chronic human lung diseases that leverage differences between the expression and roles of IL-33 in immune cells vs. non-hematopoietic cells.

## Author Contributions

LD performed the literature searches, prepared the draft versions, and edited the final version of the manuscript. YP edited the draft and final versions of the manuscript and prepared the figures. All authors contributed to the article and approved the submitted version.

## Conflict of Interest

The authors declare that the research was conducted in the absence of any commercial or financial relationships that could be construed as a potential conflict of interest.

## Abbreviations

COPD: chronic obstructive pulmonary disease
ILC2s: group 2 innate lymphoid cells
NK: natural killer
Th2: T helper 2 cell
ST2L: transmembrane ST2
sST2: soluble ST2
IL1RAcP: IL-1 receptor accessory protein
MyD88: myeloid differentiation primary response protein 88
IRAKs: IL-1R-associated kinases
TNF: tumor necrosis factor
TRAF6: TNF receptor associated factor-6
MAPK: mitogen-activated protein kinases
ERK: extracellular signal-regulated kinases
JNK: c-Jun N-terminal kinases
IκB: inhibitor of κB
NFκB: nuclear factor kappa-light-chain-enhancer of activated B cells
OVA: ovalbumin
HUVECs: human umbilical vein endothelial cells
VEGF: vascular endothelial growth factor
ASM: airway smooth muscle
AHR: airway hyperresponsiveness
PCLS: precision-cut lung slices.
